# 2-[1-(Methyl­sulfan­yl)naphtho[2,1-*b*]furan-2-yl]acetic acid

**DOI:** 10.1107/S1600536808000901

**Published:** 2008-01-16

**Authors:** Hong Dae Choi, Pil Ja Seo, Byeng Wha Son, Uk Lee

**Affiliations:** a Department of Chemistry, Dongeui University, San 24 Kaya-dong Busanjin-gu, Busan 614-714, Republic of Korea; b Department of Chemistry, Pukyong National University, 599-1 Daeyeon 3-dong, Nam-gu, Busan 608-737, Republic of Korea

## Abstract

The title compound, C_15_H_12_O_3_S, was prepared by alkaline hydrolysis of ethyl 2-{1-(methyl­sulfan­yl)naphtho[2,1-*b*]furan-2-yl}acetate. The crystal structure is stabilized by CH_2_—H⋯π inter­actions between the methyl H atoms of the methyl­sulfanyl substituent and the central benzene ring of the naphthofuran system, and by inversion-related inter­molecular O—H⋯O hydrogen bonds between the carboxyl groups.

## Related literature

For the crystal structures of similar 1-(methyl­sulfan­yl)naphtho[2,1-*b*]furan compounds, see: Choi *et al.* (2006 ▶, 2007 ▶).
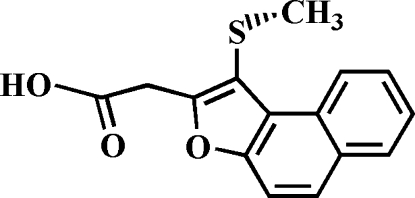


## Experimental

### 

#### Crystal data

C_15_H_12_O_3_S
*M* *_r_* = 272.32
Monoclinic, 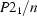

*a* = 4.989 (2) Å
*b* = 14.265 (5) Å
*c* = 18.344 (7) Å
β = 90.18 (2)°
*V* = 1305.5 (9) Å^3^
*Z* = 4
Mo *K*α radiation
μ = 0.25 mm^−1^
*T* = 296 (2) K
0.45 × 0.28 × 0.09 mm

#### Data collection

Bruker SMART CCD diffractometer
Absorption correction: none
8459 measured reflections
2209 independent reflections
1130 reflections with *I* > 2σ(*I*)
*R*_int_ = 0.115

#### Refinement

*R*[*F* ^2^ > 2σ(*F* ^2^)] = 0.051
*wR*(*F* ^2^) = 0.224
*S* = 1.24
2209 reflections
174 parameters
H-atom parameters constrained
Δρ_max_ = 1.09 e Å^−3^
Δρ_min_ = −1.45 e Å^−3^

### 

Data collection: *SMART* (Bruker, 2001 ▶); cell refinement: *SAINT* (Bruker, 2001 ▶); data reduction: *SAINT*; program(s) used to solve structure: *SHELXS97* (Sheldrick, 2008 ▶); program(s) used to refine structure: *SHELXL97* (Sheldrick, 2008 ▶); molecular graphics: *ORTEP-3* (Farrugia, 1997 ▶) and *DIAMOND* (Brandenburg, 1998 ▶); software used to prepare material for publication: *SHELXL97*.

## Supplementary Material

Crystal structure: contains datablocks global, I. DOI: 10.1107/S1600536808000901/rk2074sup1.cif

Structure factors: contains datablocks I. DOI: 10.1107/S1600536808000901/rk2074Isup2.hkl

Additional supplementary materials:  crystallographic information; 3D view; checkCIF report

## Figures and Tables

**Table 1:** Hydrogen-bond geometry (Å, °) *Cg* is the centroid of the C2/C3/C8–C11 benzene ring.

*D*—H⋯*A*	*D*—H	H⋯*A*	*D*⋯*A*	*D*—H⋯*A*
O3—H3⋯O2^i^	0.82	1.91	2.711 (4)	167
C15—H15*B*⋯*Cg*^ii^	0.96	3.03	3.949 (3)	161

Symmetry codes: (i) 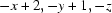; (ii) 
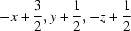
.

**Fig. 1.**
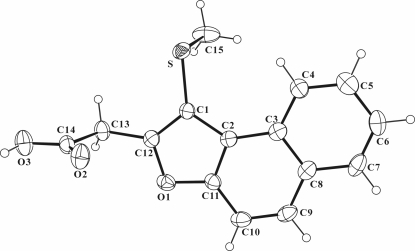
The molecular structure of the title compound with the atom numbering scheme. Displacement ellipsoides are drawn at the 30% probability level. H atoms are presented as a small spheres of arbitrary radius.

**Fig. 2.**
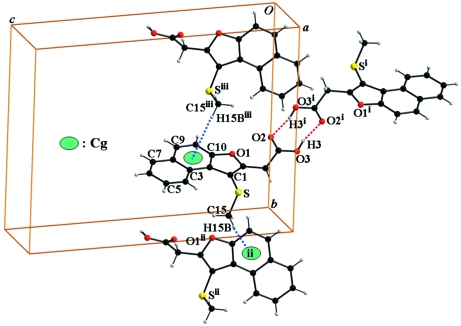
The C–H···π interaction and O–H···O hydrogen bond (dotted lines) in the title compound. Cg denotes ring centroids. [Symmetry code: (i) -x + 2, -y + 1, -z; (ii) -x + 3/2, y + 1/2, -z + 1/2; (iii) -x + 3/2, y - 1/2, -z + 1/2.]

## supplementary crystallographic information

### Comment

As part of our ongoing studies on the synthesis and structure of 1-(methylsulfanyl)naphtho[2,1-*b*]furan derivatives, we have recently described 7-bromo-1-methylsulfanyl-2-phenylnaphtho[2,1-*b*]furan (Choi *et al.*, 2006) and 2-(4-bromophenyl)-1-(methylsulfanyl)naphtho[2,1-*b*]furan (Choi *et al.*, 2007). Herein we report the molecular and crystal structure of the title compound, 2-{1-(methylsulfanyl)naphtho[2,1-*b*]furan-2-yl}acetic acid (Fig. 1).

The naphthofuran unit is essentially planar, with a mean deviation of 0.017Å from the least-squares plane defined by the thirteen constituent atoms. The crystal packing (Fig. 2) is stabilized by CH_2_—H···π interactions, with a C15—H15B···*Cg* separation of 3.03Å (*Cg* is the centroid of the C2/C3/C8/C9/C10/C11 benzene ring; symmetry code as in Fig. 2). Classical inversion- related O3–H3···O2^i^ hydrogen bonds link the carboxyl groups of adjacent molecules (Table and Fig. 2).

### Experimental

Ethyl 2-{1-(methylsulfanyl)naphtho[2,1-*b*]furan-2-yl}acetate (600 mg, 2.0 mmol) was added to a solution of potassium hydroxide (561 mg, 10.0 mmol) in water (20 ml) and methanol (20 ml). The mixture was refluxed for 4 h and then cooled. Water was added, and the solution was washed with chloroform. The aqueous layer was acidified to pH 1 with concentrated hydrochloric acid and then extracted with chloroform, dried over magnesium sulfate, filtered and concentrated under vacuum. The residue was purified by column chromatography (hexane/ethyl-acetate, 1:1 *v*/*v*) to afford the title compound as a colourless solid [yield 82%, m.p. 436–437 K; *R*_f_ = 0.62 (hexane/ethyl-acetate, 1:1 *v*/*v*)]. Single crystals suitable for X-ray diffraction were prepared by slow evaporation of a dilute solution of the title compound in diisopropyl ether at room temperature.

Spectroscopic analysis: ^1^H NMR (CDCl_3_, 400 MHz) δ 2.39 (s, 3H), 4.17 (s, 2H), 7.49–7.54 (m, 1H), 7.60–7.67 (m, 2H), 7.74 (d, J = 9.16 Hz, 1H), 7.95 (d, J = 7.68 Hz, 1H), 9.18 (d, J = 8.44 Hz, 1H), 11.02 (s, 1H); EI—MS 272 [*M*^+^].

### Refinement

All H atoms were positioned geometrically and refined using a riding model, with C–H = 0.93 Å for aromatic H atoms, C–H = 0.96 Å for methyl H atoms, C–H = 0.97 Å for methylene H atoms, and O–H = 0.82 Å, respectively, and with *U*_iso_(H) = 1.2*U*_eq_(C) for aromatic and methylene H atoms, *U*_iso_(H) = 1.5*U*_eq_(C) for methyl H atoms and *U*_iso_(H) = 1.5*U*_eq_(O) for caroxylic H atom.

The highest peak (1.088 e^.^Å^-3^) in the difference map is 0.97Å from S and the largest hole (-1.449 e^.^Å^-3^) is 0.21Å from S.

### Crystal data

**Table:**

C_15_H_12_O_3_S	*F*_000_ = 568
*M**_r_* = 272.32	*D*_x_ = 1.385 Mg m^−^^3^
Monoclinic, *P*2_1_/*n*	Melting point = 436–437 K
Hall symbol: -P 2yn	Mo *K*α radiation λ = 0.71073 Å
*a* = 4.989 (2) Å	Cell parameters from 3886 reflections
*b* = 14.265 (5) Å	θ = 2.2–27.9º
*c* = 18.344 (7) Å	µ = 0.25 mm^−^^1^
β = 90.18 (2)º	*T* = 296 (2) K
*V* = 1305.5 (9) Å^3^	Plate, silver
*Z* = 4	0.45 × 0.28 × 0.09 mm

### Data collection

**Table:**

Bruker SMART CCD diffractometer	2209 independent reflections
Radiation source: fine-focus sealed tube	1130 reflections with *I* > 2σ(*I*)
Monochromator: graphite	*R*_int_ = 0.115
Detector resolution: 10.0 pixels mm^-1^	θ_max_ = 25.5º
*T* = 296(2) K	θ_min_ = 1.8º
φ and ω scans	*h* = −6→4
Absorption correction: none	*k* = −17→17
8459 measured reflections	*l* = −22→21

### Refinement

**Table:**

Refinement on *F*^2^	Secondary atom site location: difference Fourier map
Least-squares matrix: full	Hydrogen site location: inferred from neighbouring sites
*R*[*F*^2^ > 2σ(*F*^2^)] = 0.051	H-atom parameters constrained
*wR*(*F*^2^) = 0.224	*w* = 1/[σ^2^(*F*_o_^2^) + (0.1148*P*)^2^] where *P* = (*F*_o_^2^ + 2*F*_c_^2^)/3
*S* = 1.24	(Δ/σ)_max_ < 0.001
2209 reflections	Δρ_max_ = 1.09 e Å^−^^3^
174 parameters	Δρ_min_ = −1.45 e Å^−^^3^
Primary atom site location: structure-invariant direct methods	Extinction correction: none

### Special details

**Table:**

Geometry. The s.u.'s (except the s.u.'s in the dihedral angle between two l.s. planes) are estimated using the full covariance matrix. The cell s.u.'s are taken into account individually in the estimation of s.u.'s in distances, angles and torsion angles; correlations between s.u.'s in cell parameters are only used when they are defined by crystal symmetry. An approximate (isotropic) treatment of cell s.u.'s is used for estimating s.u.'s involving l.s. planes.
Refinement. Refinement of *F*^2^ against ALL reflections. The weighted *R*-factor *wR* and goodness of fit *S* are based on *F*^2^, conventional *R*-factors *R* are based on *F*, with *F* set to zero for negative *F*^2^. The threshold expression of *F*^2^ > 2σ(*F*^2^) is used only for calculating *R*-factors(gt) *etc*. and is not relevant to the choice of reflections for refinement. *R*-factors based on *F*^2^ are statistically about twice as large as those based on *F*, and *R*-factors based on ALL data will be even larger.

### Fractional atomic coordinates and isotropic or equivalent isotropic displacement parameters (Å^2^)

**Table:**

	*x*	*y*	*z*	*U*_iso_*/*U*_eq_	
S	0.7770 (3)	0.81307 (6)	0.19046 (5)	0.0475 (5)	
O1	1.1260 (7)	0.57778 (15)	0.25355 (14)	0.0476 (9)	
O2	0.8997 (7)	0.5410 (2)	0.08670 (16)	0.0591 (9)	
O3	1.2812 (7)	0.5715 (2)	0.02196 (17)	0.0637 (10)	
H3	1.2206	0.5315	−0.0056	0.096*	
C1	0.8881 (9)	0.7104 (2)	0.23497 (18)	0.0371 (10)	
C2	0.8115 (10)	0.6749 (2)	0.30727 (18)	0.0378 (11)	
C3	0.6288 (10)	0.7029 (2)	0.36497 (19)	0.0417 (11)	
C4	0.4543 (10)	0.7812 (3)	0.3628 (2)	0.0487 (12)	
H4	0.4531	0.8190	0.3215	0.058*	
C5	0.2867 (13)	0.8034 (3)	0.4195 (3)	0.0629 (15)	
H5	0.1742	0.8551	0.4156	0.076*	
C6	0.2828 (11)	0.7483 (3)	0.4842 (2)	0.0604 (13)	
H6	0.1703	0.7641	0.5226	0.073*	
C7	0.4471 (13)	0.6718 (3)	0.4889 (2)	0.0589 (16)	
H7	0.4450	0.6353	0.5310	0.071*	
C8	0.6247 (11)	0.6465 (2)	0.4295 (2)	0.0459 (12)	
C9	0.7927 (11)	0.5650 (2)	0.4337 (2)	0.0529 (13)	
H9	0.7865	0.5284	0.4757	0.063*	
C10	0.9638 (12)	0.5385 (2)	0.3780 (2)	0.0520 (13)	
H10	1.0721	0.4857	0.3820	0.062*	
C11	0.9664 (10)	0.5950 (2)	0.31502 (19)	0.0402 (10)	
C12	1.0731 (10)	0.6499 (2)	0.20589 (19)	0.0403 (11)	
C13	1.2273 (9)	0.6483 (2)	0.1351 (2)	0.0457 (12)	
H13A	1.4123	0.6325	0.1458	0.055*	
H13B	1.2257	0.7110	0.1146	0.055*	
C14	1.1236 (10)	0.5807 (2)	0.0778 (2)	0.0423 (11)	
C15	0.9473 (11)	0.9017 (2)	0.2426 (3)	0.0734 (17)	
H15A	0.8677	0.9062	0.2900	0.110*	
H15B	0.9321	0.9609	0.2181	0.110*	
H15C	1.1331	0.8853	0.2475	0.110*	

### Atomic displacement parameters (Å^2^)

**Table:**

	*U*^11^	*U*^22^	*U*^33^	*U*^12^	*U*^13^	*U*^23^
S	0.0597 (9)	0.0476 (5)	0.0353 (6)	0.0031 (6)	−0.0090 (7)	0.0046 (3)
O1	0.0544 (19)	0.0403 (10)	0.0479 (15)	0.0044 (16)	−0.011 (2)	−0.0052 (10)
O2	0.0561 (19)	0.0729 (17)	0.0483 (17)	−0.019 (2)	0.002 (2)	−0.0139 (14)
O3	0.058 (2)	0.0800 (19)	0.0536 (18)	−0.010 (2)	0.000 (2)	−0.0234 (15)
C1	0.041 (2)	0.0385 (13)	0.0316 (16)	−0.001 (2)	−0.005 (2)	−0.0046 (12)
C2	0.041 (3)	0.0375 (14)	0.0344 (19)	−0.004 (2)	−0.009 (3)	−0.0031 (12)
C3	0.046 (3)	0.0429 (14)	0.0355 (18)	−0.009 (2)	−0.012 (3)	−0.0047 (13)
C4	0.048 (3)	0.0574 (18)	0.041 (2)	0.006 (3)	−0.008 (3)	−0.0054 (15)
C5	0.059 (3)	0.069 (2)	0.062 (3)	0.008 (3)	−0.015 (4)	−0.015 (2)
C6	0.053 (3)	0.079 (3)	0.049 (2)	−0.006 (4)	0.008 (3)	−0.019 (2)
C7	0.079 (4)	0.065 (2)	0.0334 (19)	−0.026 (3)	0.006 (3)	−0.0025 (15)
C8	0.052 (3)	0.0489 (16)	0.0368 (18)	−0.015 (2)	−0.008 (3)	−0.0018 (13)
C9	0.072 (4)	0.0483 (16)	0.0379 (19)	−0.008 (3)	−0.012 (3)	0.0079 (13)
C10	0.068 (3)	0.0392 (14)	0.049 (2)	0.003 (2)	−0.016 (3)	0.0025 (14)
C11	0.044 (2)	0.0384 (13)	0.0385 (18)	0.002 (2)	−0.006 (3)	−0.0056 (13)
C12	0.044 (3)	0.0403 (14)	0.0361 (18)	−0.006 (2)	−0.005 (3)	−0.0047 (12)
C13	0.044 (3)	0.0508 (17)	0.042 (2)	−0.008 (2)	0.003 (3)	−0.0109 (14)
C14	0.045 (3)	0.0474 (16)	0.0343 (18)	0.004 (2)	0.000 (3)	−0.0064 (14)
C15	0.082 (4)	0.0414 (16)	0.096 (3)	−0.006 (3)	−0.040 (4)	0.0067 (18)

### Geometric parameters (Å, °)

**Table:**

S—C1	1.766 (3)	C6—C7	1.368 (7)
S—C15	1.797 (4)	C6—H6	0.9300
O1—C12	1.375 (4)	C7—C8	1.453 (7)
O1—C11	1.404 (5)	C7—H7	0.9300
O2—C14	1.263 (5)	C8—C9	1.435 (6)
O3—C14	1.300 (5)	C9—C10	1.386 (7)
O3—H3	0.8200	C9—H9	0.9300
C1—C12	1.372 (6)	C10—C11	1.410 (5)
C1—C2	1.471 (5)	C10—H10	0.9300
C2—C11	1.384 (5)	C12—C13	1.512 (6)
C2—C3	1.455 (6)	C13—C14	1.516 (4)
C3—C4	1.417 (6)	C13—H13A	0.9700
C3—C8	1.431 (5)	C13—H13B	0.9700
C4—C5	1.373 (7)	C15—H15A	0.9600
C4—H4	0.9300	C15—H15B	0.9600
C5—C6	1.423 (7)	C15—H15C	0.9600
C5—H5	0.9300		
			
C1—S—C15	100.99 (18)	C10—C9—C8	122.8 (3)
C12—O1—C11	105.7 (3)	C10—C9—H9	118.6
C14—O3—H3	109.5	C8—C9—H9	118.6
C12—C1—C2	108.2 (3)	C9—C10—C11	117.1 (4)
C12—C1—S	123.5 (3)	C9—C10—H10	121.4
C2—C1—S	128.3 (3)	C11—C10—H10	121.4
C11—C2—C3	120.1 (3)	C2—C11—O1	112.3 (3)
C11—C2—C1	103.2 (4)	C2—C11—C10	123.3 (4)
C3—C2—C1	136.7 (3)	O1—C11—C10	124.4 (4)
C4—C3—C8	117.1 (4)	C1—C12—O1	110.6 (4)
C4—C3—C2	125.6 (3)	C1—C12—C13	133.5 (3)
C8—C3—C2	117.3 (4)	O1—C12—C13	115.9 (3)
C5—C4—C3	122.4 (4)	C12—C13—C14	115.6 (4)
C5—C4—H4	118.8	C12—C13—H13A	108.4
C3—C4—H4	118.8	C14—C13—H13A	108.4
C4—C5—C6	121.0 (5)	C12—C13—H13B	108.4
C4—C5—H5	119.5	C14—C13—H13B	108.4
C6—C5—H5	119.5	H13A—C13—H13B	107.4
C7—C6—C5	118.9 (5)	O2—C14—O3	126.5 (3)
C7—C6—H6	120.5	O2—C14—C13	119.7 (4)
C5—C6—H6	120.5	O3—C14—C13	113.8 (4)
C6—C7—C8	121.2 (4)	S—C15—H15A	109.5
C6—C7—H7	119.4	S—C15—H15B	109.5
C8—C7—H7	119.4	H15A—C15—H15B	109.5
C3—C8—C9	119.4 (4)	S—C15—H15C	109.5
C3—C8—C7	119.4 (4)	H15A—C15—H15C	109.5
C9—C8—C7	121.2 (4)	H15B—C15—H15C	109.5
			
C15—S—C1—C12	−106.1 (4)	C3—C8—C9—C10	0.9 (6)
C15—S—C1—C2	72.4 (4)	C7—C8—C9—C10	179.8 (4)
C12—C1—C2—C11	1.0 (4)	C8—C9—C10—C11	−0.5 (6)
S—C1—C2—C11	−177.7 (3)	C3—C2—C11—O1	178.7 (3)
C12—C1—C2—C3	−178.8 (4)	C1—C2—C11—O1	−1.1 (4)
S—C1—C2—C3	2.5 (7)	C3—C2—C11—C10	−1.8 (6)
C11—C2—C3—C4	−178.0 (4)	C1—C2—C11—C10	178.4 (4)
C1—C2—C3—C4	1.7 (7)	C12—O1—C11—C2	0.9 (4)
C11—C2—C3—C8	2.0 (5)	C12—O1—C11—C10	−178.6 (4)
C1—C2—C3—C8	−178.3 (4)	C9—C10—C11—C2	1.0 (6)
C8—C3—C4—C5	0.0 (6)	C9—C10—C11—O1	−179.5 (3)
C2—C3—C4—C5	180.0 (4)	C2—C1—C12—O1	−0.5 (4)
C3—C4—C5—C6	0.6 (6)	S—C1—C12—O1	178.3 (3)
C4—C5—C6—C7	−0.7 (7)	C2—C1—C12—C13	−179.2 (4)
C5—C6—C7—C8	0.3 (7)	S—C1—C12—C13	−0.4 (6)
C4—C3—C8—C9	178.5 (4)	C11—O1—C12—C1	−0.2 (4)
C2—C3—C8—C9	−1.5 (5)	C11—O1—C12—C13	178.7 (3)
C4—C3—C8—C7	−0.4 (5)	C1—C12—C13—C14	−102.7 (5)
C2—C3—C8—C7	179.6 (4)	O1—C12—C13—C14	78.6 (4)
C6—C7—C8—C3	0.3 (6)	C12—C13—C14—O2	10.5 (5)
C6—C7—C8—C9	−178.6 (4)	C12—C13—C14—O3	−170.9 (3)

### Hydrogen-bond geometry (Å, °)

**Table:**

*D*—H···*A*	*D*—H	H···*A*	*D*···*A*	*D*—H···*A*
O3—H3···O2^i^	0.82	1.91	2.711 (4)	167
C15—H15B···Cg^ii^	0.96	3.03	3.949 (3)	161

Symmetry codes: (i) −*x*+2, −*y*+1, −*z*; (ii) −*x*+3/2, *y*+1/2, −*z*+1/2.
